# The Pathobiology of H7N3 Low and High Pathogenicity Avian Influenza Viruses from the United States Outbreak in 2020 Differs between Turkeys and Chickens

**DOI:** 10.3390/v13091851

**Published:** 2021-09-16

**Authors:** Miriã F. Criado, Christina M. Leyson, Sungsu Youk, Suzanne DeBlois, Tim Olivier, Mary Lea Killian, Mia L. Torchetti, Darren J. Parris, Erica Spackman, Darrell R. Kapczynski, David L. Suarez, David E. Swayne, Mary J. Pantin-Jackwood

**Affiliations:** 1 Exotic and Emerging Avian Viral Diseases Research Unit, Southeast Poultry Research Laboratory, U.S. National Poultry Research Center, Agricultural Research Service, U.S. Department of Agriculture, Athens, GA 30605, USA; mcriado@uga.edu (M.F.C.); christina.leyson@usda.gov (C.M.L.); sungsu.youk@usda.gov (S.Y.); suzanne.deblois@usda.gov (S.D.); tim.olivier@usda.gov (T.O.); dparri11@gmail.com (D.J.P.); erica.spackman@usda.gov (E.S.); darrell.kapczynski@usda.gov (D.R.K.); david.suarez@usda.gov (D.L.S.); david.swayne@usda.gov (D.E.S.); 2 National Veterinary Services Laboratories, Animal and Plant Health Inspection Service, U.S. Department of Agriculture, Ames, IA 50010, USA; mary.l.killian@usda.gov (M.L.K.); mia.kim.torchetti@usda.gov (M.L.T.)

**Keywords:** H7N3, high pathogenicity avian influenza, low pathogenicity avian influenza viruses, chickens, turkeys, infectivity, pathogenicity, transmission

## Abstract

An outbreak caused by H7N3 low pathogenicity avian influenza virus (LPAIV) occurred in commercial turkey farms in the states of North Carolina (NC) and South Carolina (SC), United States in March of 2020. Subsequently, H7N3 high pathogenicity avian influenza virus (HPAIV) was detected on a turkey farm in SC. The infectivity, transmissibility, and pathogenicity of the H7N3 HPAIV and two LPAIV isolates, including one with a deletion in the neuraminidase (NA) protein stalk, were studied in turkeys and chickens. High infectivity [<2 log_10_ 50% bird infectious dose (BID_50_)] and transmission to birds exposed by direct contact were observed with the HPAIV in turkeys. In contrast, the HPAIV dose to infect chickens was higher than for turkeys (3.7 log_10_ BID_50_), and no transmission was observed. Similarly, higher infectivity (<2–2.5 log_10_ BID_50_) and transmissibility were observed with the H7N3 LPAIVs in turkeys compared to chickens, which required higher virus doses to become infected (5.4–5.7 log_10_ BID_50_). The LPAIV with the NA stalk deletion was more infectious in turkeys but did not have enhanced infectivity in chickens. These results show clear differences in the pathobiology of AIVs in turkeys and chickens and corroborate the high susceptibility of turkeys to both LPAIV and HPAIV infections.

## 1. Introduction

Avian influenza viruses (AIV) are type A influenza viruses belonging to the Orthomyxoviridae family and are classified into subtypes based on the two viral surface glycoproteins: the hemagglutinin (HA) and the neuraminidase (NA). The virus is further classified as either low pathogenicity (LP) or high pathogenicity (HP) based on lethality in chickens or sequence determination of the HA cleavage site of H5 and H7 subtypes that is consistent with HP viruses [1,2]. Wild aquatic birds are the natural reservoir of AIVs; viruses from these species are usually of the LP phenotype, and infections are asymptomatic [2]. Periodically, these LPAIVs transmit from wild to domestic birds, resulting in subclinical infections, mild respiratory disease, and/or drops in egg production [3]. After circulating in chickens or turkeys, H5 and H7 LPAIVs have mutated on numerous occasions to HPAIV, causing poultry outbreaks worldwide [2,4].

Between 1955 and 2019, forty-two unique lineages of HPAIV outbreaks have been reported around the world [4]. In the Americas in the last 20 years, H7 HPAIV outbreaks in poultry have occurred in Chile (H7N3) in 2002 [5], in Canada (H7N3) in 2004 [6] and 2007 [7], and in Mexico in 2012 (H7N3), with the latter virus becoming endemic in this country [8,9,10]. In the United States (US) in 2016, H7N8 HPAIV and its LPAIV precursor were detected in a turkey flock in Indiana [11]. In 2017, H7N9 HPAIV was identified in two broiler breeder farms in Tennessee, with the precursor LPAIV found in multiple broiler breeder farms and backyard poultry in Tennessee and neighboring states [12].

In March 2020, an outbreak of H7N3 LPAIV occurred in turkey farms in North Carolina and South Carolina [13]. In April 2020, an H7N3 HPAIV was also detected in one of the turkey premises. Around 400,000 birds were depopulated to control the outbreak [13]. Whole genome sequencing of the viruses from this outbreak showed that the H7N3 gene segments have a North American wild bird origin and are genetically distinct from the viruses previously identified in the 2016 and 2017 US H7N8 and H7N9 outbreaks, respectively [13]. Although they are the same HA and NA subtype, the 2020 H7N3 viruses are genetically distinct from the Mexican H7N3 HPAIVs that have been circulating in poultry since 2012. The 2020 US H7N3 virus isolates were all highly similar to each other, indicating a single introduction of a wild bird H7N3 LPAIV into turkeys with subsequent mutation to HPAIV [13]. Two notable genetic changes were identified among the virus isolates: a multibasic cleavage site in the HA gene present in the HPAIV isolates and a deletion in the NA stalk region found in some of the LPAIV isolates from the outbreak [13]. Deletions in the NA stalk have been previously associated with adaptation to gallinaceous species [14,15,16,17].

In an effort to improve the control of LPAIV and HPAIV in poultry and increase understanding of the pathobiology of these viruses in gallinaceous species, in this study, we characterized the infectivity, transmissibility, and pathogenicity of two H7N3 LPAIVs, one of them with the NA stalk deletion and one of the HPAIV isolates from the 2020 outbreak in the two most important poultry species, turkeys and chickens.

## 2. Materials and Methods

### 2.1. Viruses

Two LPAIV isolates, A/turkey/South Carolina/20-008394-1/2020 (H7N3) (LPAIV-1) (GenBank accession number MT444368-MT444375) and A/turkey/North Carolina/20-008425-1/2020 (H7N3) (LPAIV-2) (GenBank accession number MT444287-MT444294), and one HPAIV, A/turkey/South Carolina/20-010561-006/2020 (H7N3) (HPAIV) (GenBank accession number MT444408-MT444415), were used in this study. The viruses were isolated from an active surveillance testing program in North Carolina and South Carolina, US, during March–April 2020, and sequenced [13]. The virus isolates were kindly provided by the National Veterinary Services Laboratories (NVSL) of the US Department of Agriculture, Animal and Plant Health Inspection Service (USDA-APHIS). The working virus stocks were propagated and titrated by allantoic sac inoculation of 9- to 10-day-old embryonated chicken eggs (ECE) by standard methods [18]. The LPAIVs differed mainly by a 66 nucleotide (nt) deletion in the NA stalk region of LPAIV-2. The HPAIV had a 27 nt insertion in the HA cleavage site compared to the LPAIVs and did not have the NA stalk deletion [13]. Next-generation sequencing conducted in our laboratory [19] determined that the HPAIV isolate was a mix of LPAIV and HPAIV. To obtain a preparation with only HPAIV, the first ECE passage of the field HPAIV isolate was passaged in 10-day-old ECEs and then passaged a second time in 14-day-old ECEs, which is a procedure that has been shown to select for HPAIV [20,21]. Brain tissue harvested from embryos after the second ECE passage were homogenized and subjected to another passage in 14-day-old ECEs. This last passage in ECE was used as inoculum. Sequence reads from randomly amplified RNA in the last ECE passage were assembled to the sequence of the original HPAIV isolate from the outbreak using BWA-MEM [22,23], and subsequently, variants were determined using Lofreq [24]. No variants lacking the multibasic cleavage site (MBCS) were detected, demonstrating that only viruses with the MBCS were in the HPAIV preparation. Brain–heart infusion (BHI) broth (Becton Dickinson and Company, Sparks, MD, USA) was used to dilute the virus stocks to the appropriate dose. Full genome sequencing was conducted of the inoculum used for all the three viruses and sequences compared to that of the original published (GenBank) field viruses. No changes were found in LPAIV-1 and LPAIV-2. The HPAIV used as inoculum had four amino acid changes when compared to the published GenBank sequence (D44N in matrix (M), T251S in neuraminidase (NA), D680N and L648M in polymerase basic protein (PB2) segments). Experiments were performed in a biosafety level-3 enhanced (BSL-3E) facility in accordance with procedures approved by the U.S. National Poultry Research Center (USNPRC) Institutional Biosecurity Committee, Agricultural Research Service (ARS), USDA.

### 2.2. Animals and Housing

One-day-old turkeys (*Meleagris gallopavo*) were provided by a commercial producer and reared at the USNPRC until three weeks of age. Four-week-old specific-pathogen free (SPF) White leghorn chickens (*Gallus gallus*) were obtained from the USNPRC in-house flocks. Birds were transferred to the animal biosafety level 3 enhanced (ABSL3E) facilities at the USNPRC, where each experimental group was housed in self-contained isolation units ventilated under negative pressure and inlet and outlet HEPA filtration. Turkeys and chickens had ad libitum access to food and water throughout the experiment. Housing and experimental procedures were reviewed and approved by the USNPRC Institutional Animal Care and Use Committee (IACUC).

### 2.3. Experimental Design

A total of 91 chickens and 61 turkeys were used in this study. The experimental design was similar to previous studies [25,26,27]. A reduced number of turkeys was used compared to chickens because of bird availability and the number of birds that could be housed per isolator. Ten birds of each species were bled prior to virus inoculation to confirm the absence of AIV antibodies by ELISA using the IDEXX AI MultiS-Screen ELISA kit (Westbrook, ME, USA) according to the manufacturer’s protocol. Birds were divided into groups, and each group was inoculated intrachoanally with one virus at the appropriate dose to determine the 50% bird infectious dose (BID_50_) for each virus in each bird species. The virus doses were initially intended to be 2 (low dose), 4 (medium dose), or 6 (high dose) log_10_ 50% egg infective dose (EID_50_) in 0.1 mL per bird as in previous studies, but back titers of the LPAIVs inocula confirmed that the low dose was 2 log_10_, the medium dose was 3 log_10_, and the high dose was 5 log_10_ EID_50_. A separate identical experiment was repeated for the LPAIVs only in chickens using the dose of 6 log_10_ EID_50_ to obtain an endpoint to determine the BID_50_ for these viruses.

In all experiments, a group of sham-inoculated birds were inoculated intrachoanally with 0.1 mL of sterile allantoic fluid diluted 1:300 in BHI medium. In addition, to evaluate the transmissibility of each virus, two or three naïve birds from the same species were added to each dose group 24 h after inoculation (contact-exposed birds).

Oropharyngeal (OP) and cloacal (CL) swabs were collected at 12, 24 and 36 h post-inoculation, followed by 2, 3, 4, 7, 10 and 14 days post-inoculation (dpi). All swabs were placed in brain–heart infusion (BHI) medium with penicillin (2000 units/mL; Sigma Aldrich, St. Louis, MO, USA), gentamicin (200 μg/mL, Sigma Aldrich; St. Louis, MO, USA) and amphotericin B (5 μg/mL, Sigma Aldrich; St. Louis, MO, USA) and stored at −80 °C to determine virus shed titers. Two turkeys from the groups that received the high dose of HPAIV (6 log_10_ EID_50_) and three chickens from each of the three high-dose groups for each virus were euthanized and necropsied at 2 dpi. Brain, heart, spleen, lung, and muscle tissues were collected from these birds and stored at −80 °C for virus detection and quantification. All birds were observed daily for clinical signs and mortality from 0 to 14 dpi (direct inoculates) or 0 to 13 days post-contact (dpc). Birds showing severe clinical signs including severe listlessness, neurological signs, respiratory distress, or inability to eat or drink were euthanized and counted as dead the next day for mean death time (MDT) calculations. At day 14, surviving birds were bled and euthanized.

Sera collected from all surviving birds was used to evaluate infection status by antibody levels using the hemagglutination inhibition (HI) assay. HI assays were performed using standard methods and homologous antigen [28]. Seroconversion was also confirmed by the ELISA test using the IDEXX AI MultiS-Screen ELISA kit (Westbrook, ME, USA). HI titers less than 3 log_2_ GMT were considered negative. A signal-to-negative ratio (S/N) of greater than or equal to 0.5 was considered negative for the ELISA tests. HI assay and ELISA results were consistent with each other. The mean bird infectious dose (BID_50_) for each virus was calculated by the Reed–Muench method [29], using the criteria that birds were considered infected if they shed detectable levels of virus at any time and/or were positive for antibody at the end of the study.

### 2.4. Viral Titration in Swabs and Tissues

Swab and tissue samples were processed for quantitative real-time RT-PCR (qRT-PCR) to determine viral titers. We used a standard protocol that demonstrated the high correlation between qRT-PCR and the infectious titer determined in ECE as previously described [30]. Briefly, for oropharyngeal and cloacal swab samples, total RNA was extracted using MagMAX™–96 AI/ND Viral RNA Isolation Kit^®^ (Ambion Inc./Thermo Fisher Scientific; Grand Island, NY, USA) according to the manufacturer’s protocol. For tissues, samples were homogenized and resuspended in BHI media to a 10% (*w*/*v*) solution, and the total RNA was extracted from the homogenates using Trizol LS reagent (Invitrogen/Thermo Fisher Scientific; Grand Island, NY, USA) and chloroform (Life Technologies/Thermo Fisher Scientific, Carlsbad, CA, USA) according to the manufacturer’s protocol. The resulting aqueous supernatants from tissue RNA extracts were purified using an RNA Clean and Concentrator kit (Zymo, Irvine, CA, USA), quantified by NanoDrop™ 1000 Spectrophotometer (Thermo Fisher Scientific) following the manufacturer’s instructions, and diluted with Tris-EDTA buffer (10 mM Tris, 0.1 mM EDTA, pH 7.5) to obtain 50 ng/µL.

qRT-PCR was performed with the AgPath-ID One-Step RT-PCR kit (Ambion/Thermo Scientific; Grand Island, NY, USA) using a 7500 FAST real-time PCR system (Applied Biosystems, Foster City, CA, USA) and matrix-specific primers and a probe as previously described [27]. The standard curves for viral RNA quantification were established with 10-fold dilutions of RNAs extracted from the same titrated stocks used for inoculation. Results were reported as EID_50_/mL or EID_50_/g equivalents, and the lower limit of detection was set based on each standard curve. The lower limit of detection was 1.8 log_10_ EID_50_/mL for LPAIV-1, 1.5 log_10_ EID_50_/mL for LPAIV-2, and 1.5 log_10_ EID_50_/mL for HPAIV. For statistical purposes, qRT-PCR negative samples were given a value of 0.1 log_10_ EID_50_/mL below the test limit of detection.

### 2.5. Statistical Analysis

Statistical analyses were performed using Prism 8 (GraphPad Software, San Diego, CA, USA; version 8.4.3). Virus RNA titers between OP and CL swabs were statistically compared at each time point using two-way ANOVA with Sidak’s multiple comparisons. Statistical differences in the area under the curve of the plot between viral RNA titers, and time points were analyzed using one-way ANOVA with Sidak’s multiple comparisons test. Those *p* values < 0.05 were considered significant.

### 2.6. Sequence Analyses

Phylogenetic trees were adapted using the same set of sequences from a previous study [13]. Whole genome sequences (GenBank numbers MT444183-350 and MT444352-415) were obtained from the nucleotide database of the National Center for Biotechnology Information (NCBI). For each isolate, eight segments were concatenated in order of decreasing segment length. Concatenated sequences were aligned using MAFFT [31], and maximum likelihood trees were generated using RaxML as implemented in Geneious Prime 2019.2.3 (Biomatters Ltd.; Auckland, New Zealand) and the CIPRES Science Gateway [32]. Pairwise sequence identities among the concatenated genomes of the three virus isolates were calculated in Geneious Prime 2019.2.3. Additionally, pairwise differences among the genomes of the three isolates were identified and annotated using custom scripts in Python 3.8.5 (Conda version 4.10.1, https://anaconda.com; accessed on 3 September 2020). A sequence difference was considered nonsynonymous if the change results in an amino acid change in at least one protein encoded in the corresponding segment.

## 3. Results

### 3.1. Infectivity, Transmission, and Pathogenicity of the H7N3 LPAIVs in Turkeys and Chickens

Birds were considered infected if they shed virus and/or seroconverted by the end of the study (14 dpi or 13 dpc). Results are presented in Table 1. None of the turkeys inoculated with the low dose (2 log_10_ EID_50_) of LPAIV-1 became infected; however, all turkeys were infected in the medium (3 log_10_ EID_50_) and high dose (5 log_10_ EID_50_) groups, resulting in a BID_50_ of 2.5 log_10_ EID_50_ (Table 1). Contact turkeys in the medium and high-dose groups of LPAIV-1 were also infected. All turkeys in the LPAIV-2 groups, including contacts, were infected, resulting in a BID_50_ of <2 log_10_ EID_50_ (Table 1).

All chickens inoculated with the highest dose (6 log_10_ EID_50_) of LPAIV-1 and LPAIV-2 were infected. Only one chicken inoculated with 5 log_10_ EID_50_ of LPAIV-1, and one chicken from the groups inoculated with 3 or 5 log_10_ EID_50_ of LPAIV-2, respectively, were also infected (Table 1). The BID_50_ was 5.7 log_10_ EID_50_ for LPAIV-1 and 5.4 log_10_ EID_50_ for LPAIV-2. Only one contact-exposed chicken in each of the LPAIV-1 and LPAIV-2 highest dose (6 log_10_ EID_50_) groups was infected (Table 1).

No or mild clinical signs (mild infraorbital swelling) were observed in the turkeys infected with either LPAIV or the contacts in these groups. However, one turkey inoculated with 3 log_10_ EID_50_ of LPAIV-2 and a contact from the same group were found dead at 11 dpi and 3 dpc, respectively. Since these turkeys, or any other turkeys in this group, showed no clinical signs, and LPAIV was confirmed by sequencing the swab samples from these birds (no HPAIV), we concluded that the cause of death was not related to the LPAIV infection, since LPAIVs do not cause mortality if not complicated with other factors. No gross lesions were identified in the dead turkeys, and the cause of death was not determined. No clinical signs were observed in the chickens infected with the LPAIVs, and no gross lesions were observed in the birds necropsied at 2 dpi.

### 3.2. Infectivity, Transmission, and Pathogenicity of the H7N3 HPAIV in Turkeys and Chickens

All turkeys inoculated with the HPAIV at all three challenge doses, and the contacts in those groups, were infected and died, with mean death times (MDTs) between 2 and 2.4 days for inoculated turkeys and 3 days post-exposure for contacts (Table 1). The BID_50_ for this virus in inoculated turkeys was <2 log_10_ EID_50_. The turkeys showed neurological signs (tremors, ataxia), lethargy, green diarrhea, mild periorbital swelling, and conjunctivitis. One turkey had blood in wing feather shafts. The gross lesions observed in the two turkeys necropsied at 2 dpi included congested internal organs, enlarged heart, and moderate splenomegaly with parenchymal mottling.

All chickens inoculated with the medium and high doses (4 and 6 log_10_ EID_50_) of H7N3 HPAIV were infected, but only one chicken from the low dose (2 log_10_ EID_50_) group was infected. Thus, the BID_50_ for H7N3 HPAIV in chickens was 3.7 log_10_ EID_50_. None of the contact chickens in this group were infected (Table 1). All infected chickens died, with MDTs between 1 and 1.6 days, which was about half a day shorter than for turkeys. In contrast to turkeys, which showed some clinical signs before death or euthanasia, most chickens died without showing clinical signs (peracute disease). Ruffled feathers, lethargy, anorexia, prostration, mild periorbital swelling, green diarrhea, and cyanotic combs were observed in the rest. The gross lesions observed in the necropsied chickens included congested internal organs and petechial hemorrhage in cecal tonsils.

### 3.3. Viral Shedding and Virus Detection in Tissues

**Figure 1:**
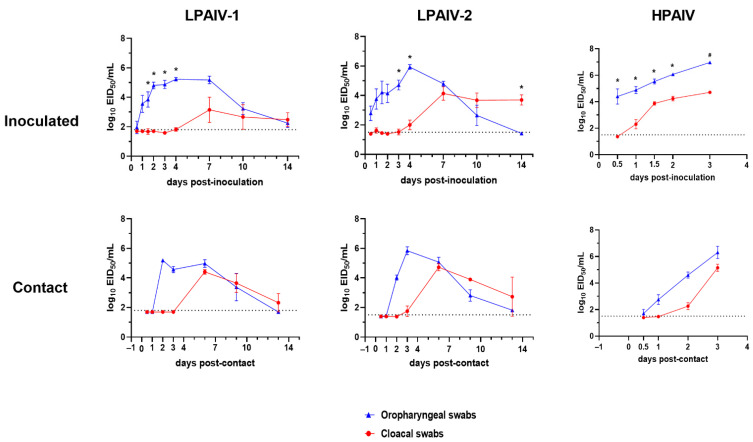
Virus shedding for turkeys inoculated with 5 log_10_ EID_50_ of LPAIV-1 and LPAIV-2 or 6 log_10_ EID_50_ of HPAIV and their respective contact-exposed turkeys. Virus titers from oropharyngeal (OP) (blue) and cloacal (CL) (red) swabs were determined by qRT-PCR. Dotted lines indicate the limit of detection for each virus. Asterisks (*) indicate statistically significant difference between OP and CL virus titers (one-way ANOVA with Sidak’s multiple comparisons test; *p* < 0.05). Only two contact birds were available; thus, no statistical test was performed for these groups.

**Figure 2:**
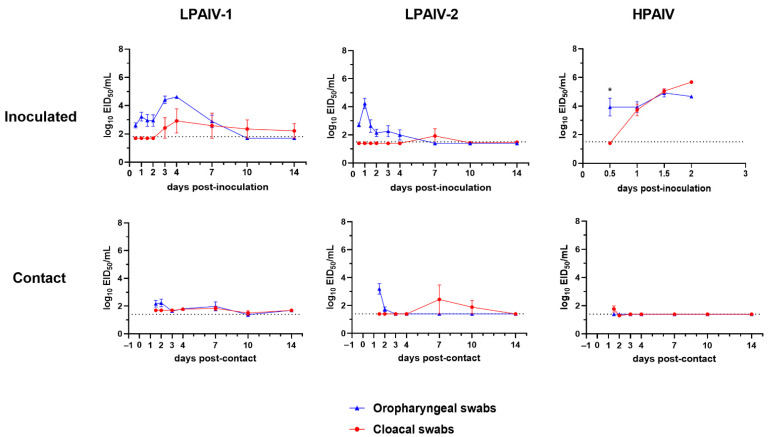
Virus shedding for chickens inoculated with 6 log_10_ EID_50_ of LPAIV-1, LPAIV-2, or HPAIV. Virus titers from oropharyngeal (OP) (blue) and cloacal (CL) (red) swabs were determined by qRT-PCR. Dotted lines indicate the limit of detection for each virus. Asterisks (*) indicate statistically significant difference between OP and CL virus titers (one-way ANOVA with Sidak’s multiple comparisons test; *p* < 0.05).

Oropharyngeal (OP) and cloacal (CL) virus shedding was evaluated in inoculated and contact-exposed turkeys and chickens by qRT-PCR (Table 1). Figure 1 and Figure 2 show the shedding results for the groups inoculated with the highest dose of the viruses. No viral RNA was detected in OP or CL swabs from turkeys inoculated with 2 log_10_ EID_50_ of LPAIV-1. However, all turkeys that received higher doses of LPAIV-1 (3 and 5 log_10_ EID_50_) shed virus, predominantly by the OP route, with some turkeys shedding virus by both routes after 7 dpi. All turkeys inoculated with LPAIV-2, independent of the inoculation dose, shed virus by the OP and CL route, with higher titers shed by the OP route at 3 and 4 dpi. Interestingly, at 14 dpi, the turkeys were shedding significantly higher titers by the CL route compared to the OP route (*p* < 0.05) (Figure 1). The peak of OP virus shedding for both LPAIVs was between 3 and 7 dpi.

**Table 1:** Infectivity, lethality, and transmission results from turkeys and chickens inoculated with the US 2020 H7N3 LPAIVs and HPAIV.

Bird Species	Virus	Dose (log_10_ EID_50_)	Inoculated					Contact Exposed			
			No. of Birds Shedding Virus/Total	No. of Dead Birds/Total (MDT) ^1^	No. of Birds HI Positive/Total (Mean HI Titer) ^2^	No. of Birds Infected/Total ^3^	BID_50_ (log_10_) ^4^	No. of Birds Shedding Virus/Total	No. of Dead Birds/Total (MDT)	No. of Birds HI Positive/Total (Mean HI Titer) ^2^	No. of Birds Infected/Total ^3^
**Turkeys**	LPAIV-1	2	0/4	0/4	0/4 (<3.0)	0/4	2.5	na	na	na	na
		3	5/5	0/5	5/5 (9.4)	5/5		2/2	0/2	2/2 (10)	2/2
		5	5/5	0/5	5/5 (9.4)	5/5		2/2	0/2	2/2 (10)	2/2
	LPAIV-2	2	5/5	0/5	5/5 (8.0)	5/5	<2	na	na	na	na
		3	5/5	1/5 ^5^	4/4 (6.5)	5/5		2/2	1/2 ^5^	1/1 (8.0)	2/2
		5	5/5	0/5	5/5 (7.6)	5/5		2/2	0/2	2/2 (6.0)	2/2
	HPAIV	2	5/5	5/5 (2.4)	na	5/5	<2	na	na	na	na
		4	5/5	5/5 (2.2)	na	5/5		2/2	2/2 (3)	na	2/2
		6	5/5	5/5 (2.0)	na	5/5		2/2	2/2 (3)	na	2/2
**Chickens**	LPAIV-1	2	0/5	0/5	0/5 (<3.0)	0/5	5.7	0/3	0/3	0/3 (<3.0)	0/3
		3	0/5	0/5	0/5 (<3.0)	0/5		0/3	0/3	0/3 (<3.0)	0/3
		5	1/8	0/8	0/5 ^6,7^ (<3.0)	1/8		0/3	0/3	0/3 (<3.0)	0/3
		6	8/8	0/8	5/5 ^6^ (6.0)	8/8		3/3	0/3	1/3 (5.0)	3/3
	LPAIV-2	2	0/5	0/5	0/5 (<3.0)	0/5	5.4	0/3	0/3	0/3 (<3.0)	0/3
		3	1/5	0/5	1/5 (3.0)	1/5		0/3	0/3	0/3 (<3.0)	0/3
		5	1/8	0/8	0/5 ^6,7^ (3.0)	1/8		0/3	0/3	0/3 (<3.0)	0/3
		6	8/8	0/8	5/5 ^6^ (4.5)	8/8		3/3	0/3	1/3 (7.0)	3/3
	HPAIV	2	1/5	1/5 (1)	0/5 (<3.0)	1/5	3.7	0/3	0/3	0/3 (<3.0)	0/3
		4	5/5	5/5 (1.6)	na	5/5		0/3	0/3	0/3 (<3.0)	0/3
		6	8/8	8/8 (1.6)	na	8/8		0/3	0/3	0/3 (<3.0)	0/3

^1^ MDT, mean death time, number of dead birds × dpi/total dead birds expressed as dpi (days post-inoculation), or dpc, (days post-contact). ^2^ Mean HI titers for birds that survived (14 dpi or 13 dpc). Titer expressed in geometric mean titers (GMT log_2_). Samples with titers < 3.0 log_2_ GMT were considered negative. ^3^ Inoculated or contact birds were considered infected if they shed virus and/or were positive for antibodies at 14 dpi or 13 dpc. ^4^ BID_50_: 50% bird infectious dose. ^5^ Birds died of undetermined causes. qRT-PCR positive for at least two time points. ^6^ The number of birds is reduced due to necropsy at 2 dpi. ^7^ One of the necropsied birds were qRT-PCR positive for at least two time points but serology was unavailable because the bird was euthanized at 2 dpi. na: not applicable.

High viral titers were shed by all HPAIV-inoculated turkeys regardless of the dose received. Virus was detected at 12 h post-inoculation (hpi) from turkeys inoculated with 6 log_10_ EID_50_ (Figure 1). All turkeys shed HPAIV by the CL route, but virus titers were significantly lower than what were observed in OP swabs (Figure 1).

Independent of the virus and dose used, contact-exposed turkeys had similar shedding patterns to those observed in the inoculated turkeys (Figure 1), with virus detected in the OP samples at 2 dpc and subsequently in both OP and CL samples in the following days (Figure 1).

In the chickens inoculated with the LPAIV-1 and LPAIV-2, OP virus shedding was detected in all the birds that received the highest virus dose (6 log_10_ EID_50_); but low or no virus was detected in CL samples (Figure 2 and Table 1). One chicken in the group inoculated with 5 log_10_ EID_50_ of LPAIV-1 and one chicken in each group that received 3 or 5 log_10_ EID_50_ of LPAIV-2 also shed virus (Table 1). The chickens inoculated with 5 log_10_ EID_50_ of LPAIV-1 shed higher virus titers for more days compared to chickens inoculated with LPAIV-2 (Figure 2). All chickens inoculated with 4 log_10_ EID_50_ (Table 1) and 6 log_10_ EID_50_ (Table 1 and Figure 2) of the HPAIV shed high virus titers by the OP and CL routes. Only one of five chickens inoculated with 2 log_10_ EID_50_ of the HPAIV shed at 12 hpi and was euthanized due to severe clinical signs (Table 1).

Contact chickens that became infected in the groups inoculated with 6 log_10_ EID_50_ of the LPAIVs shed low virus titers by both routes (Figure 2). However, chickens exposed by contact to the HPAIV-inoculated chickens did not shed any detectable virus (Figure 2). These contact chickens also did not show clinical signs or seroconverted, so they were not considered infected (Table 1).

**Figure 3:**
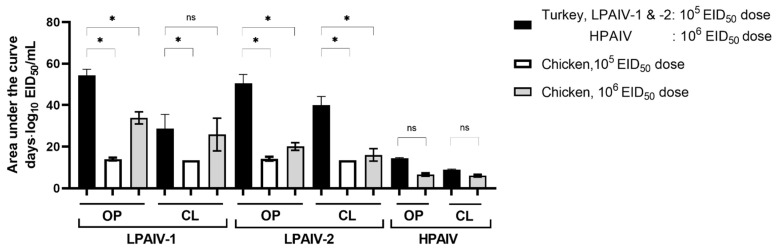
Area under the curve calculation for qRT-PCR data to quantify the amount and duration of virus shed. Asterisks (*) indicate statistically significant difference between indicated groups (one-way ANOVA with Sidak’s multiple comparisons test; *p* < 0.05).

To compare the amount and duration of virus shedding for all three isolates, areas under the curve (AUC) were calculated using qRT-PCR data from groups inoculated with the highest doses of the isolates (5 or 6 log_10_ EID_50_) (Figure 3). Except for CL viral shedding by chickens inoculated with 6 log_10_ EID_50_ of LPAIV-2, turkeys have a significantly higher AUC values for virus shedding compared to chickens for LPAIV-1 and LPAIV-2 but not HPAIV (Figure 3). For turkeys, AUCs of OP and CL shedding were significantly different between HPAIV and LPAIV-1 or LPAIV-2. No differences in OP or CL shedding for turkeys were observed between LPAIV-1 and LPAIV-2. For chickens given a 6 log_10_ EID_50_ dose, AUCs for OP shedding were significantly different between HPAIV and LPAIV-1 or LPAIV-2, while AUCs for CL shedding were only statistically significant between HPAIV and LPAIV-1. Statistically significant differences in AUCs between chickens inoculated with 6 log_10_ EID_50_ of LPAIV-1 and LPAIV-2 were only observed for OP shedding but not with CL shedding. Thus, in chickens, AUCs of OP shedding was higher in LPAIV-1 compared to that of LPAIV-2 or HPAIV (Figure 3, Supplementary Table S1).

The brain, heart, lung, muscle, and spleen were collected from necropsied birds for viral quantification by qRT-PCR (Table 2). We analyzed tissues from chickens inoculated with the H7N3 LPAIV-1, LPAIV-2, and the HPAIV. As a result of the reduced number of turkeys, only tissues from HPAIV-inoculated turkeys were collected and examined. In general, results obtained for viral detection in turkey and chicken tissues were consistent with the mortality data, with high virus titers found in most tissues from birds infected with the HPAIV. Turkeys had high titers of virus (ranging from 5.7 to 7.7 log_10_ EID_50_) in all tissues analyzed. Most tissues collected from the HPAIV-infected chickens also had high virus titers, except for one of the chickens from the low-dose group, which had high virus titers (7.4 log_10_ EID_50_) in the heart and no or low titers in the other tissues, and the chicken from the high-dose group, which had high virus titers in all tissues except the heart (Table 2). Very low or no virus was detected in tissues from the chickens inoculated with the LPAIVs (1.62 ± 0.1 log_10_ EID_50_; *p* < 0.05).

### 3.4. Sequence Comparisons of the H7N3 Viruses

**Figure 4:**
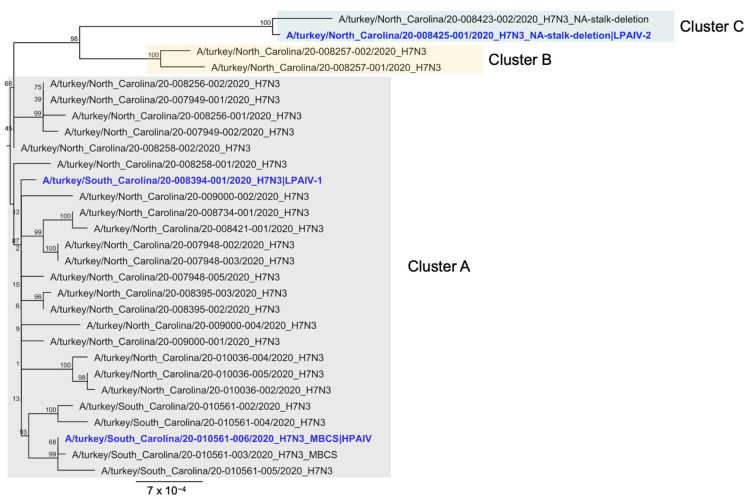
Phylogenetic tree of H7N3 virus isolates from the 2020 H7N3 AI outbreak in North and South Carolina. The isolates used as inoculum in this study are depicted in blue. All eight gene segments from each isolate were concatenated and subsequently used to construct the phylogenetic tree. Adopted from a previous study [13]. MBCS next to the isolate name indicates that a multibasic cleavage site is found in its HA gene. Bootstrap values are reported at the nodes. Scale bar unit is in nucleotide substitutions per site.

Phylogenetic trees of concatenated genome sequences from the H7N3 outbreak in North and South Carolina were reconstructed as previously reported [13] (Figure 4). All outbreak sequences were highly related to each other with about 99% pairwise sequence identities in each segment. Thus, the outbreak was likely caused by a single introduction from a wild bird [13]. Moreover, phylogenetic trees of each segment were congruent with the phylogenetic trees constructed from concatenated sequences.

**Figure 5:**
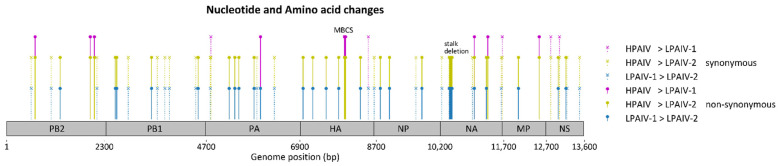
Genomic changes found between the H7N3 viruses used in this study as inoculum. Pairwise comparisons between each pair of viruses were examined. Synonymous and non-synonymous nucleotide changes are mapped onto the concatenated AIV genome. MBCS = multibasic cleavage site.

The LPAIV-1 and the HPAIV viruses used in this study belong to the main cluster of outbreak isolates, which is called Cluster A as in [13]. The LPAIV-2 belongs to a distinct cluster of isolates, Cluster C, which branched out early during the outbreak and had a relatively long branch length and is relatively distant to Cluster A [13]. Isolates with the NA stalk deletion, including LPAIV-2, are exclusively found in Cluster C. Pairwise whole genome sequence comparison of the H7N3 inoculum (Table 3) were also consistent with these observations in that LPAIV-1 and HPAIV are more closely related compared to LPAIV-2. Specific nucleotide sequence differences among the isolates were additionally identified and mapped along the genome (Figure 5 and Table 4). Thirty-six amino acid changes were found out of the fifty-seven nucleotide changes enumerated among all pairwise comparisons of isolates characterized *in vivo*. The major amino acid differences found were the NA stalk deletion in LPAIV-2 and the insertion of the multibasic cleavage site in HPAIV. Other notable changes were R98K, I353V in the nucleoprotein (NP), and G345R in the NA, which have been previously associated with changes in the pathobiology of AIVs in gallinaceous species (Table 4).

## 4. Discussion

In our laboratory, we routinely conduct standardized studies evaluating the pathobiology of AIVs in different avian species as part of the basic characterization of novel isolates [9,15,27,40,41,42,43,44,45,46,47,48,49,50,51]. These previous studies, as well as this one, provide essential information on the epidemiology of the AIVs and have informed models used to identify what type of samples and when to collect them for optimal virus detection during an outbreak. In this study, we compared the infectivity, transmissibility, and pathogenicity in turkeys and chickens of two LPAIVs, differing by a 66-nucleotide deletion in the NA stalk, and one HPAIV isolate from the H7N3 outbreak in turkeys in North Carolina and South Carolina, US, in 2020. A previous study showed that these H7N3 viruses derived from North American wild bird-origin AIVs and that they are distinct from other recent AIVs causing outbreaks in poultry in the US [13], specifically the H7N8 and H7N9 viruses from outbreaks in Indiana (2016) [11] and Tennessee (2017) [12], respectively, and the H7N3 HPAIV that has been circulating in Mexico since 2012. The 2020 H7N3 HPAIV has a 27-nucleotide insertion that appears derived from turkey host cellular 28S rRNA [13]. Whole genome sequencing determined that the two HPAIV variants isolated from the outbreak were a mix of LPAIV and HPAIV, suggesting that the mutation was caught early and the HPAIV was restricted to a single turkey premise [13]. The limited circulation of the HPAIV is supported by AIV surveillance of the poultry in the area. Interestingly, the insertion at the HA cleavage site is identical to the one found in the 2017 H7N9 HPAIV from the 2017 Tennessee poultry outbreak [12].

Although AIVs have been isolated from hundreds of bird species, the natural reservoirs of the virus are considered to be wild aquatic birds [52,53]. Chickens and turkeys are not natural hosts for AIV, and many wild waterfowl viruses will not easily infect and transmit in chickens and turkeys [25,54]. AIVs from wild birds once introduced into gallinaceous species can quickly adapt to the new host [15,25]. However, chickens and turkeys are not equally susceptible to the same isolates. The 50% bird infectious dose (BID_50_), a good measurement of host adaptation, may differ by 100 to 1000-fold between chickens and turkeys [25]. Turkeys appear to be more susceptible to AIV infection than chickens for many AIV isolates from wild waterfowl and poultry [15,27,55,56,57].

HPAIV causes a severe systemic disease with high mortality in chickens, turkeys, and other gallinaceous species [2,3]. LPAIV typically causes mild to moderate respiratory disease and can interrupt egg production in laying hens and turkey breeders [1]. However, in well-managed birds, LPAIV infection can be subclinical, which in commercial poultry may be problematic because the virus can circulate undetected with the risk of mutating and becoming HPAIV. Therefore, it is crucial to continue to conduct surveillance for AIV in poultry. The viruses causing the 2020 H7N3 outbreak were detected quickly, which permitted rapid control and eradication.

All turkeys inoculated with the 2020 H7N3 HPAIV became infected and died within 2 days, resulting in a BID_50_ of less than 2 log_10_ EID_50_. In addition, all contact turkeys became infected and died. Although the chickens infected with the HPAIV died within two days, the BID_50_ was higher (3.7 log_10_ EID_50_), and the virus did not transmit and infect contact-exposed chickens. These results clearly show the greater susceptibility and transmissibility of 2020 H7N3 HPAIV for turkeys than chickens, as has been previously reported in other HPAIV studies [14,25,27,58]. The H7N8 HPAIV from the 2016 outbreak in turkeys [27] also had a BID_50_ below 2 log_10_ EID_50_ and was transmitted to all contact turkeys, and chickens were also less susceptible with a BID_50_ of 3.2 log_10_ EID_50_ and no contact transmission. Interestingly, chickens infected with the 2017 H7N9 HPAIV outbreak were more susceptible to infection [43], the virus having a BID_50_ below 2 log_10_ EID_50_, but the virus still poorly transmitted to contact-exposed chickens [43]. The 2017 H7N9 LPAIV likely persisted longer, based on the wider geographic detections, in the chicken populations having more opportunity to adapt to this host.

Although the hemagglutinin (HA) protein has a significant influence on the pathogenesis of AIVs and is the major determinant of the HPAI phenotype, the neuraminidase (NA) is also involved in virus fitness and immune evasion in the host population [1,2]. The HA and NA are constantly under selective pressure due to the location of these proteins on the envelope of the virus. The two LPAIV isolates used in our study differed in the length of NA stalk region [13], with a 66 nucleotide deletion in the NA of the LPAIV-2 compared to LPAIV-1. A short NA stalk has been previously observed in other AIVs including H2N2, H5N1, H6N1, H7N1, H7N9, H7N3 and H9N2 subtypes [34,35,59,60,61,62,63]. Deletions in the NA stalk have been associated with poultry adaptation [14,15,16,17,64], outbreaks in the field [34], and experimental virus passage in gallinaceous birds [37,38]. Other studies showed that influenza viruses with different NA stalk lengths have different in vitro growth characteristics and plaque size [39,65,66] and differences in pathogenesis in ducks and chickens [39,63]. A study demonstrated that an amino acid deletion in the NA stalk can remove potential glycosylation sites, which may interfere in the protein’s function due to possible changes in the structure and consequently affect the immune response [67].

We analyzed the impact of the NA stalk deletion by comparing two 2020 H7N3 LPAIV isolates with and without such deletion. Our results demonstrate that both H7N3 LPAIV isolates could infect directly inoculated turkeys and transmit to contact-exposed turkeys. However, the virus with the NA deletion (LPAIV-2) was more infectious (BID_50_ of <2 log_10_ EID_50_) than the one without (LPAIV-1) (BID_50_ of 2.5 log_10_ EID_50_), which could indicate that this virus is better adapted to turkeys. The LPAIVs had similar viral shedding patterns to what was observed with the 2016 H7N8 LPAIV in turkeys, with virus shed for many days by both the OP and CL routes [27]. The extensive cloacal virus shedding, which was not observed in the chickens, could in part explain the better transmissibility of these viruses in turkeys, since higher amounts of virus shed into the environment could facilitate transmission. Moreover, the slightly higher infectivity of LPAIV-2 shows that potentially less LPAIV-2 is required to infect a turkey, thereby enabling more efficient transmission given the same amount of virus present in the environment.

Similar data demonstrating the high susceptibility of turkeys to H7 LPAIVs have been previously published [15,27,43,58]. In chickens, the LPAIV doses required to infect chickens were higher than that was needed for turkeys. For both LPAIVs, all chickens only became infected when given the high virus dose (6 log_10_ EID_50_), and limited transmission was observed. Although HPAIV is shed by the OP and CL route, the LPAIVs were shed in the highest quantity from the OP route in chickens. Additionally, lower AUCs of LPAIV shedding were generally observed in chickens compared to turkeys. Previous H7 LPAIVs from the US [27,43] when examined in chickens had similar BID_50_ as well as patterns of virus shedding, and they did not transmit to contacts, indicating that these viruses were probably not well adapted to chickens. Interestingly, the presence of the NA stalk deletion did not increase the infectivity of the LPAIV-2 in chickens as seen with the turkeys. Indeed, the LPAIV-1 replicated better than the LPAIV-2, based on virus shedding, indicating that for this virus, the effect of the NA stalk deletion might be species specific. These observations are consistent with the fact that the 2020 H7N3 outbreak occurred in turkey premises and thus, efficient replication in the turkey host was likely selected for in the H7N3 viruses as the outbreak continued.

Aside from the multibasic cleavage site in the HA and the NA stalk deletion, thirty-six amino acid differences were found among the three 2020 H7N3 viruses, two of which have been previously reported in similar studies. One of them is the R98K change in the nucleoprotein (NP) of the HPAIV. This change was also observed with the Goose/Guangdong lineage H5N2 HPAIVs causing the outbreak in poultry in the US in 2015, suggesting that R98K may be associated with adaptation of HPAIVs in poultry [44]. Another change, the G345R substitution in the NA observed in the LPAIV-2, was also found in H7N3 HPAIV isolates from chickens in Mexico, with the glycine residue observed in the earlier 2012 isolate, whereas the arginine residue was observed in a 2016 isolate [10]. It remains to be determined if the other amino acid changes have a role in adaptation of these viruses in poultry. Since the outbreak was rapidly contained, the viruses had limited opportunity to accumulate changes that could further affect the pathobiology of the viruses.

Analyses from all gene segments of the H7N3 LPAIV and HPAIV isolates from the 2020 outbreak in the US [13] suggest that the precursor for these viruses most likely emerged from wild waterfowl in the Mississippi flyway with occasional spread during migration. Spillover of North American lineage H7 subtype AIV from wild birds into poultry has occurred several times [6,7,11,12,68], highlighting the importance of constant AIV surveillance in wild birds and poultry, and enhanced biosecurity for poultry during periods of wild bird migration.

In conclusion, our results showed that the H7N3 2020 LPAIVs and the HPAIV were more infectious in turkeys than in chickens and were efficiently transmitted to contact turkeys but not chickens, corroborating the high susceptibility of turkeys to AIV infections. This high susceptibility of turkeys to AIV infection, coupled with high titers and duration of virus shed, favored the spread of these AIVs in turkeys. The LPAIV-2 was more infectious to turkeys than LPAIV-1, which was possibly due to the 66 nucleotide deletion at the NA stalk region, but this effect was not seen in chickens. Additional experiments using reverse genetics could help determine possible markers of host adaptation and virulence and better associate the NA stalk length with the differences observed in pathogenesis, infectivity, and transmission between different species. Furthermore, there is a need to understand why changes only occur in specific LPAIV precursors that favor the emergence of HPAIV. Finally, this knowledge will enhance our ability to predict and implement strategies to prevent potential AIV outbreaks.

## Figures and Tables

**Table 2:** Virus detection in tissues of turkeys and chickens inoculated with the 2020 H7N3 HPAIV. Tissues were taken from three birds euthanized at 2 dpi and virus titer was determined by qRT-PCR.

Species	Bird Number	Dose Received (log_10_ EID_50_)	Virus Titer (log_10_ EID_50_/g)				
			Brain	Heart	Lung	Muscle	Spleen
Turkey	1	6	6.5	7.7	6.3	5.9	6.0
	2	6	6.7	7.0	6.2	5.8	6.0
	3	6	6.6	7.7	6.2	5.7	6.3
Chicken	1	2	2.5	7.4	-	2.4	-
	2	4	6.8	7.5	6.4	7.0	6.8
	3	6 *	7.0	1.8	6.3	7.0	6.7

- = negative. * Seven out of the eight chickens that were given a dose of 6 log_10_ EID_50_ died or were euthanized at 1.5 dpi. Thus, two chickens from other groups (dose 2 and 4 log_10_ EID_50_) were chosen for necropsy.

**Table 3:** Pairwise comparison of concatenated whole genomes of US 2020 H7N3 LPAI and HPAI viruses used as challenge viruses in animal experiments.

	LPAIV-1	LPAIV-2	HPAIV
LPAIV-1		99.205	99.713
LPAIV-2	99.205		98.956
HPAIV	99.713	98.956	

**Table 4:** Important amino acid sequence changes found between the US 2020 H7N3 LPAIVs and HPAIV used as inoculum for the animal experiments. na: not applicable.

LPAIV-1	LPAIV-2	HPAIV	Position	Amino Acid Change	Protein	References	Remarks
na	na	DRKSRHRRI	339–347	Insertion: DRKSRHRRI	Hemagglutinin		Multibasic cleavage site
R	R	K	98	R98K	Nucleoprotein	[33]	Change found in samples from bobwhite quail infected with a 2014 H5N2 HPAIV virus from the US 2014–2015 H5 outbreak. Also found in later 2015 virus isolates from turkeys in Minnesota.
na	LNCSDTIITYNNTVINNITTTI	na	56–77	Deletion: LNCSDTIITYNNTVINNITTTI	Neuraminidase	[16,17,34,35,36,37,38,39]	Associated with adaptation to gallinaceous species
G	R	G	345	G345R	Neuraminidase	[26]	Found in a 2016 H7N3 virus from Mexico, when compared to an earlier 2012 H7N3 virus.

## Data Availability

The data that support the findings of this study are provided in the figures and tables of the article. Additional information is available from the corresponding author upon reasonable request.

## Supplementary Materials

The following are available online at https://www.mdpi.com/article/10.3390/v13091851/s1, Table S1: Statistical comparisons between area under the curves (AUCs) of virus shedding from turkeys and chickens inoculated with H7N3 viruses as measured by qRT-PCR.
