# Supplementary material for: The Pathobiology of H7N3 Low and High Pathogenicity Avian Influenza Viruses from the United States Outbreak in 2020 Differs between Turkeys and Chickens
Source: Viruses. 2021 Sep 16;13(9):1851. doi: 10.3390/v13091851 (PMC8472980; doi:10.3390/v13091851)
Supplement: Supplementary file 1 [file viruses-13-01851-s001.zip › viruses-1367729-supplementary.pdf]

Supplementary Table S1. Statistical comparisons between area under the curves (AUCs) of virus shedding from turkeys and chickens inoculated with H7N3 viruses as measured by qRT-PCR. ns= not significant.

Species	Swabs	Virus 1	Dose 1	Virus 2	Dose 2	95% confidence interval of the difference	Summary	Adjusted *p* Value
Turkey	OP	LPAIV-1	5	HPAIV	6	25.09 to 54.77	****	<0.0001
		LPAIV-2	5	HPAIV	6	21.35 to 51.03	****	<0.0001
		LPAIV-1	5	LPAIV-2	5	-11.10 to 18.58	ns	0.9845
	CL	LPAIV-1	5	HPAIV	6	4.898 to 34.57	**	0.0033
		LPAIV-2	5	HPAIV	6	16.33 to 46.00	****	<0.0001
		LPAIV-1	5	LPAIV-2	5	-26.27 to 3.405	ns	0.2237
Chicken	OP	LPAIV-1	6	HPAIV	6	-38.93 to -15.58	****	<0.0001
		LPAIV-2	6	HPAIV	6	-25.17 to -1.815	*	0.0126
		LPAIV-1	6	LPAIV-2	6	2.083 to 25.44	*	0.0101
	CL	LPAIV-1	6	HPAIV	6	-31.50 to -8.150	****	<0.0001
		LPAIV-2	6	HPAIV	6	-21.68 to 1.670	ns	0.1500
		LPAIV-1	6	LPAIV-2	6	-1.857 to 21.50	ns	0.1676
